# Protective Effects of *Lycium barbarum* Extracts on UVB-Induced Damage in Human Retinal Pigment Epithelial Cells Accompanied by Attenuating ROS and DNA Damage

**DOI:** 10.1155/2018/4814928

**Published:** 2018-11-07

**Authors:** Feng-Chi Hsieh, Chun-Tzu Hung, Kai-Chun Cheng, Chang-Yi Wu, Yen-Chun Chen, Yu-Jen Wu, Wangta Liu, Chien-Chih Chiu

**Affiliations:** ^1^Department of Radiology, Yuan's General Hospital, Kaohsiung 802, Taiwan; ^2^Department of Ophthalmology, Yuan's General Hospital, Kaohsiung 802, Taiwan; ^3^Department of Ophthalmology, Kaohsiung Municipal Hsiaokang Hospital, Kaohsiung 812, Taiwan; ^4^Department of Ophthalmology, Kaohsiung Medical University Hospital, 807 Kaohsiung, Taiwan; ^5^Department of Optometry, Shu-Zen Junior College of Medicine and Management, Kaohsiung 821, Taiwan; ^6^Department of Biotechnology, Kaohsiung Medical University, Kaohsiung 807, Taiwan; ^7^Department of Biological Sciences, National Sun Yat-sen University, Kaohsiung 804, Taiwan; ^8^Department of Nursing, Meiho University, Pingtung 912, Taiwan; ^9^Lipid Science and Aging Research Center, Kaohsiung Medical University, Kaohsiung 807, Taiwan; ^10^Research Center for Environment Medicine, Kaohsiung Medical University, Kaohsiung 807, Taiwan; ^11^Translational Research Center, Cancer Center, Department of Medical Research, Kaohsiung Medical University Hospital, Kaohsiung Medical University, Kaohsiung 807, Taiwan; ^12^Graduate Institute of Medicine, College of Medicine, Kaohsiung Medical University, Kaohsiung 807, Taiwan

## Abstract

The medicinal herb *Lycium barbarum* fruit has been widely used for improving and maintaining the health of the eyes in the Far East for many centuries. This study is aimed at investigating whether protective effects generated from the aqueous (LBA) and ethanol (LBE) extracts of the *L. barbarum* fruit existed against oxidative stress-induced apoptosis in human retinal pigment epithelial cells. *L. barbarum* extracts LBA and LBE exerted the activity of ROS scavenging and rescued UVB irradiation-induced growth inhibition in retinal pigment epithelial ARPE-19 cells. Compared to LBA, the ethanol extract LBE exerted a superior protective activity on UVB-induced growth arrest in ARPE-19 cells. Both *L. barbarum* extracts significantly reduced cell cycle G_2_-arrest population in ARPE-19 cells. Furthermore, the cytometer-based Annexin V/propidium iodide staining assay further showed that both *L. barbarum* extracts protected ARPE-19 cells from UVB-induced apoptosis. *L. barbarum* extracts also reduced the activation of *γ*H2AX, a sensor of DNA damage in ARPE-19 cells in a dose-responsive manner. By using Ingenuity Pathway Analysis (IPA), the bioinformatics revealed that the protective effects of both LBA and LBE extracts might be involved in three signaling pathways, especially the Toll-like receptor (TLR) pathway associated with cellular proliferation. Our study suggests that both ethanol and aqueous extracts of *L. barbarum* exhibit antioxidant activity and rescue UVB-induced apoptosis of ARPE-19 cells. Collectively, the ethanol extract exerts a superior effect on rescuing UVB-induced growth arrest of ARPE-19 compared to the aqueous extract, which might be associated with the activation of TLR signaling. Our present work will benefit the preventive strategy of herbal medicine-based vision protection for treating eye diseases such as age-related macular degeneration in the future.

## 1. Introduction

Age-related macular degeneration (AMD), a progressive macular retinal disease with degenerative changes, can be divided into atrophic and exudative, characterized by the progressive atrophy of retinal pigment epithelial (RPE) cells and the formation of choroidal neovascularization (CNV) [1]. RPE cells are located between the layers of photoreceptor cells and provide nutrition to the latter. If oxidative damage occurs in RPE cells, the breakdown of photoreceptor cells would quickly follow and visual acuity might become damaged [2].

The fruit of *Lycium barbarum* (LB) wolfberry is a traditional Chinese herbal medicine that has multiple functions in pharmacology [3] like antioxidation [4–6], antiaging [7, 8], neuroprotection [9–12], cytoprotection [13, 14], and immunomodulating [5, 15]. A previous study showed that LBP (*Lycium barbarum* polysaccharides) extracted from the fruit of *L. barbarum* might be responsible for the above biological activities [16]. LBP was also shown to exert a protective effect against oxidative damage in cells [17–20]. Based on the antioxidant activity of *L. barbarum*, many studies have demonstrated that LBP has a protective effect against oxidative injury in cells [17–20], and many studies have focused on the bioactivities of this extract of *L. barbarum*. However, the effects of the ethanol fraction of LB extracts have been little addressed by previous studies. In the study, we prepared both aqueous (LBA) and ethanol (LBE) extracts of LB and investigated the protective effects of LBA or LBE on human retinal pigment epithelial (ARPE-19) cells from UVB damage, notably proliferation inhibition and apoptosis. We also discussed the possible mechanism underlying *L. barbarum* extract-mediated protective effect on retinal pigment epithelial cells.

## 2. Materials and Methods

### 2.1. Plant Material and Extraction

A total of 500 g of dried fruits of *L. barbarum* were placed in boiling 3 L water (100°C) for 4 h according to a traditional method described as in the previous study [21]. After filtration, using Whatman no. 3 filter paper, the aqueous extract of *L. barbarum* was lyophilized. For the ethanol extracts, 500 g of dried fruits was placed in 3 L of ethanol for 3 h at 70°C. The solution was filtrated with Whatman no. 3 filter paper and then evaporated at 35°C with reduced pressure.

### 2.2. Cell Culture

Arising retinal pigment epithelia cell line-19 (ARPE-19), a monolayer of polarized epithelial cells located between the sensory retina and choriocapillaris, is differentiated and mitotically inactive under normal physiological conditions. The ARPE-19 (No. 60,383), obtained from the Bioresource Collection and Research Center (BCRC, Hsinchu, Taiwan), was grown in DMEM medium (Dulbecco's Modified Eagle's Medium, Invitrogen Corporation, Carlsbad, CA, USA) supplemented with 10% (v/v) fetal bovine serum, 100 units/mL penicillin, and 100 *μ*g/mL streptomycin in an incubator with 5% CO_2_ at 37°C. Cells were pretreated with *L. barbarum* extracts (from 0 to 200 *μ*g/mL) for 2 h; then, cells were exposed to 50 mJ/cm^2^ of UVB cultured for 24 h. The experiment of different exposing dose of UVB was performed in triplicate and repeated three times to ensure reproducibility.

### 2.3. Assessment of Cell Viability

The cell viability was assessed using a colorimetric tetrazolium 3-[4, 5-dimethylthiazol-2-yl]-2, 5 diphenyl tetrazolium bromide (MTT) assay. Briefly, 1 × 10^4^ cells were seeded into a 96-well plate, and the cells were treated with different exposing doses of UVB (from 0 to 60 mJ/cm^2^) for indicated periods (24 and 48 hr, respectively). The final concentration of MTT in each well was 0.5 mg/mL, and the cells were further incubated. Afterward, the media containing MTT were removed, and the crystal formazan was dissolved entirely with Dimethyl sulfoxide (DMSO). The absorption length of light was measured at 570 nm using a microplate reader (Thermo, Massachusetts, USA), and the relative cell viability was presented as the percentage of absorbance values in treated cells to that of control cells.

### 2.4. Assessment of Cell Cycle Distribution

The assay of flow cytometer-based propidium iodide (PI) staining was used for detecting cell cycle distribution as described previously [22]. Briefly, ARPE-19 cells were pretreated with LBA and LBE for 2 h; then, the cells were irradiated with 50 mJ/cm^2^ UVB for 24 hr. Afterward, trypsin-suspended cells were washed and ethanol-fixed. After centrifugation, the cells were stained with 10 *μ*g/mL PI (Sigma, St. Louis, MO) and 10 *μ*g/mL RNase A at room temperature in the dark. The PI-stained cells were further analyzed by FACScan, a flow cytometer (Becton-Dickinson, Mansfield, MA), and WinMDI/PC-freeware V2.9 (Joseph Trotter, La Jolla, CA, USA).

### 2.5. Assessment of Mitochondrial Membrane Potential (Δ*Ψ*m)

The changes in the mitochondrial membrane potential (Δ*Ψ*m) were assessed using a mitochondrial permeable lipophilic cationic dye 5,5,6,6-Tetrachloro-1,1,3,3-tetraethylbenzimidazolylcarbocyanine iodide (JC-1). In healthy cells, JC-1 accumulates in the mitochondria and emits red fluorescence (560 nm); however, in mitochondrial cells with changed membrane potential, the JC-1 accumulates in the cytoplasm and emits fluorescence (530 nm). ARPE-19 cells were pretreated with LBA and LBE and incubated with 10 mg/mL JC-1 at 37°C in the dark. Cells were washed twice with serum-free medium and detected using a fluorescence microscope (Olympus IX71 CTS).

### 2.6. FITC-Annexin V/PI Apoptosis Assay

The assessment of apoptotic cells was performed according to previous work [22]. Briefly, cells were pretreated with LB extracts 2 hr, respectively, prior to UVB irradiation for 24 hr. The changes of early and late apoptosis were determined using an Annexin-V-Fluorescein isothiocyanate (FITC)/PI Apoptosis Detection kit (BioVision, CA 94043, USA). Briefly, cells were resuspended in binding buffer then incubated in the dark with FITC-labeled Annexin V and propidium iodide for 15 min at room temperature; finally, the samples were diluted with phosphate-buffered saline (PBS). Flow cytometry was carried out on a FACScan instrument (FACS Calibur; Becton Dickinson, Mountain View, CA, USA), and data were processed with WinMDI/PC-software V2.9 (written by Joseph Trotter, Scripps Research Institute, La Jolla, CA, USA). Cells labeled with annexin conjugated with the FITC fluorescence were recognized as apoptotic populations.

### 2.7. Determination of Intracellular ROS

We measured the changes in intracellular ROS according to the previous work with minor modifications [23]. 5 × 10^5^ cells were seeded in a six-well plate in triplicate. The cells were pretreated with or without LBA and LBE 2 hr prior to the UVB irradiation. After 12 hr, the cells were washed twice with PBS, collected, and then further incubated with 10 *μ*M dichloro-dihydro-fluorescein diacetate probe (DCFH-DA, Molecular Probes Inc., Eugene, OR, USA) at 37°C for 30 min. The fluorescence intensity of DCFH was quantified by flow cytometry (the length of excitation is 485 nm, and the length of emission is 525 nm). The results were presented as the percentage of the control cells (100%).

### 2.8. Assessment of DNA Damage

Briefly, cells were pretreated with LB extracts 2 hr, respectively, prior to UVB irradiation. Afterward, cells were harvested and fixed with 70% ethanol at −20°C overnight; then, the cells were washed twice with BSA-T-PBS solution (1% BSA and 0.2% Triton X-100 in PBS). The cells were then incubated with 0.2 *μ*g/mL antibodies against phosphorylated-Ser^139^ H2AX (*γ*H2AX), and the secondary antibody was subsequently conjugated with Alexa Fluor 488 (Jackson Laboratory, Bar Harbor, Marine, USA). The intensity of fluorescence was measured by flow cytometry (FACSCalibur, Becton-Dickinson). The results were quantified using the software Cell Quest (Becton-Dickinson).

### 2.9. Microarray Analysis

RNA molecules were isolated using MirVana Total RNA Isolation Kit (Applied Biosystems, Foster City, CA, USA) according to the instructions of the manufacturer. The microarray and the data analysis were performed by Welgene Biotech (Taipei, Taiwan) using the SurePrint G3 GE 8 × 60 K Microarray, 8 × 60 K, AMADID 028005 (Agilent Technologies, USA [24]). The arrays were scanned with G2505C Microarray Scanner (Agilent). The information of probes on the arrays was extracted from the image data using Feature extraction 10.5.1.1 (Agilent) for quantifying signal and the intensity of background.

### 2.10. Ingenuity Pathway Analysis

The molecular functions of the unique gene analysis of the UVB-induced genes were analyzed using the software Ingenuity Pathway Analysis® (IPA, Ingenuity Systems, Redwood City, California, USA). The changed genes which met the criteria and were correlated with the biological functions of the Ingenuity Pathways Knowledge Base (Ingenuity Systems) were included.

### 2.11. Statistical Analysis

All experiments were conducted in triplicate, and the data were represented as mean ± SD. The data were subjected to an analysis of variance (ANOVA) and Duncan's multiple range tests. *p* < 0.05 was considered significant.

## 3. Results

### 3.1. UVB-Induced Cell Death in Retinal Pigment Epithelial Cells

**Figure 1:**
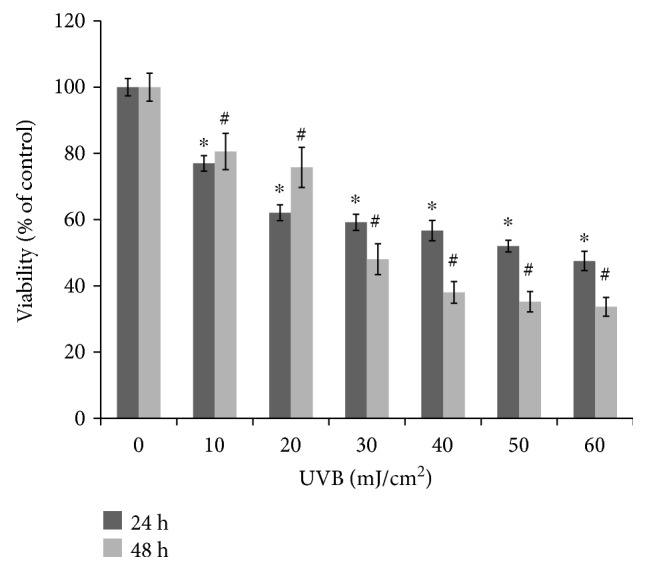
The viability of UVB irradiation on growth of ARPE-19 cells. The cells were exposed to the irradiation of UVB at indicated doses and then incubated further for 24 hr and 48 hr, respectively. The viability of cells was determined by MTT assay. The results are expressed as mean ± standard deviation (SD) (*n* = 3). The (^∗^) asterisk and (^#^) hash symbols indicate *p* < 0.05* vs.* cells without UVB irradiation for 24 hr and 48 hr, respectively.

ARPE-19 cells were exposed to UVB light with indicated doses of UVB (from 0 to 60 mJ/cm^2^, respectively) for 24 hr, and the cell viabilities were 100 ± 2.61%, 76.97 ± 2.35%, 62.08 ± 2.40%, 59.17 ± 2.43%, 56.68 ± 3.08%, 51.98 ± 1.78%, and 47.52 ± 2.92%. At 48 hr, viabilities were 100 ± 4.22%, 80.57 ± 4.48%, 75.77 ± 6.09%, 48.06 ± 4.68%, 38.02 ± 3.27%, 35.20 ± 3.08%, and 33.66 ± 2.86% (Figure 1). The results showed that the irradiation of 50 mJ/cm^2^ UVB significantly induced cell death of RPE cells.

### 3.2. *L. barbarum* Extracts Reduced UVB-Induced Cell Death in Retinal Pigment Epithelial Cells

**Figure 2:**
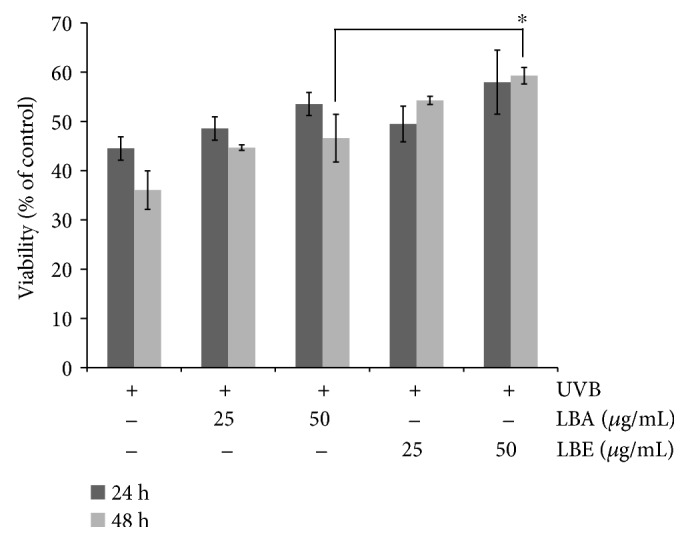
*L. barbarum* extracts rescue the viability of UVB-treated cells in an appropriate concentration. ARPE-19 cells were pretreated with either LBA or LBE for 2 hr before being exposed to UVB irradiation. The cell viability was determined by MTT assay 24 hr and 48 hr after UVB irradiation (50 mJ/cm^2^). The control group was the UVB-irradiated cells without *L. barbarum* extracts. The results are expressed as a percentage of control and are represented by mean ± SD (*n* = 3). The asterisk indicates *p* < 0.05* vs*. UVB-exposed cells without LBA and LBE pretreatments.

To evaluate whether LBA and LBE protected ARPE-19 cells against UVB-induced cell death, we detected the viability of ARPE-19 cells after UVB (50 mJ/cm^2^) incubation for 24 hr and 48 hr, with or without LBA and LBE pretreatment in 25 and 50 (*μ*g/mL). As shown in Figure 2, the viability of cells was decreased to 44.51 ± 2.38% after being exposed to UVB (50 mJ/cm^2^) for 24 hr; LBA and LBE pretreatments with a variety of concentrations from 25 *μ*g/mL to 50 *μ*g/mL for 48 hr prevented the loss of cell viability. Finally, 50 *μ*g/mL of both LBA and LBE increased the cell variety from 44.51 ± 2.38% to 53.54 ± 2.35% and from 44.51 ± 2.38% to 57.96 ± 6.50%, respectively.

### 3.3. LBA and LBE Reduce Endogenous ROS Level after Irradiated ARPE-19

**Figure 3:**
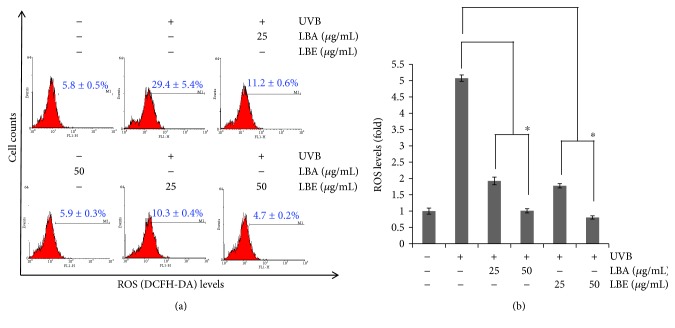
Effect of *L. barbarum* extracts on reactive oxygen species (ROS) production of ARPE19 cells after UVB irradiation exposure. (a) ARPE-19 cells pretreated with PBS or *L. barbarum* extracts LBA and LBE 25 and 50 *μ*g/mL for 2 hr. After being exposed to 50 mJ/cm^2^ UVB irradiation, cells were harvested and subjected to a flow cytometer-based DCFDA staining assay, which is oxidized by ROS to the high fluorescence of DCF for detecting the changes of H_2_O_2_ in cells. (b) The quantitative analysis of (a). The asterisk symbols indicate *p* < 0.05* vs.* UVB-irradiated cells without LBA or LBE pretreatments.

As shown in Figure 2, the pretreatments of LBA and LBE rescue the viability of ARPE-19 cells following the irradiation of UVB. Under normal conditions, reactive oxygen species (ROS) could act as a second messenger in cell signaling and regulates various biological functions [25–27]. However, excess ROS production could inhibit proliferation of cells and cause cell death [26–29]. A high level of endogenous ROS is highly correlated with the pathophysiology of retinal degeneration diseases including AMD. After exposure to UVB, levels of ROS will reach 29.4 ± 5.4%, compared to pretreatment with LBA and LBE in a high dose of 50 (*μ*g/mL) then down to 5.9 ± 0.3% and 4.7 ± 0.2%, respectively. As shown in Figure 3, LBA and LBE reduced the levels of endogenous ROS in human ARPE-19 cells after the irradiation of UVB (*p* < 0.05).

### 3.4. LBA and LBE Significantly Reduced UVB-Induced Apoptosis of ARPE-19

**Figure 4:**
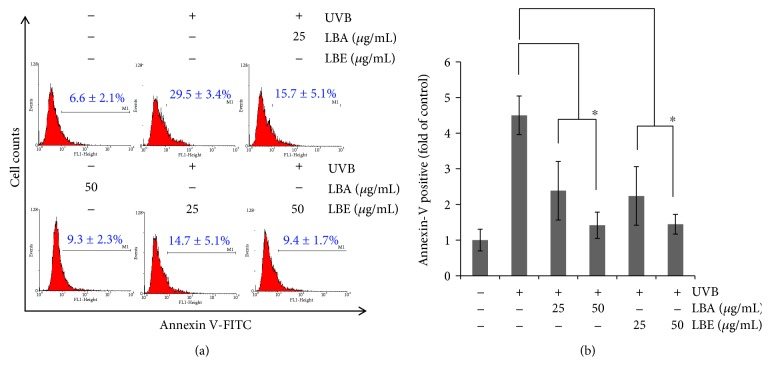
Pretreatment with *L. barbarum* extracts rescues UVB-induced apoptosis of retinal pigment epithelial cells. (a) ARPE-19 cell was pretreated with vehicle control or *L. barbarum* extracts LBA and LBE for 2 hr, respectively, prior to the irradiation of 50 mJ/cm^2^ UVB. Afterward, cells were harvested and assessed using a flow cytometer-based Annexin V staining. Results are represented as mean ± SD. (b) The quantitative analysis of (a). The asterisk indicates *p* < 0.05* vs*. UVB-irradiated cells without LBA and LBE pretreatments.

To determine whether LBA and LBE mitigated cell apoptosis in UVB-irradiated ARPE-19 cells, the cytometer-based Annexin V/PI dual staining assay was conducted. ARPE-19 cells were cultured with 0, 25, and 50 *μ*g/mL of LBA and LBE for 24 hr before being irradiated with UVB 50 mJ/cm^2^ for 24 hr. Data are represented as (mean ± SD) of five individual experiments, with *p* < 0.05 compared with control. Following UVB-induced apoptosis of ARPE-19 cells, incubation of two treatments for 24 hr demonstrated levels of apoptotic cells decreased from 29.5 ± 3.4% to 15.7 ± 5.1% and 9.3 ± 2.3% by LBA and down to 14.7 ± 5.1% and 9.4 ± 1.7% by LBE, respectively. As shown in Figure 4, the results showed that both treatments protect apoptosis in the ARPE-19 cells, suggesting the protective effects of LBA and LBE on ARPE-19 cell.

### 3.5. *L. barbarum* Extracts Attenuate UVB-Induced Loss of Mitochondrial Membrane Potential

**Figure 5:**
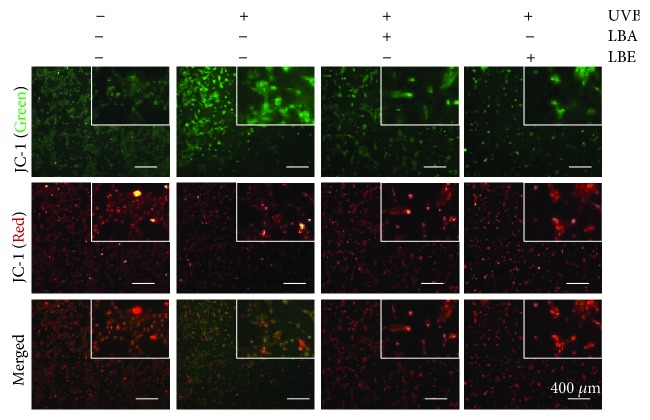
The effect of *L. barbarum* extracts on the UVB-induced loss of mitochondrial membrane potential (MMP). ARPE-19 cells pretreated with 50 *μ*g/mL LBA or LBE, respectively, prior to the irradiation of 50 mJ/cm^2^ UVB light. Afterward, the changes of MMP (Δ*Ψ*m) were detected by a fluorescence microscope-based JC-1 staining assay. The wavelengths of emission are 580 nm (red, upper panels) and 530 nm (green, lower panels). Scale bars: 400 *μ*m.

Mitochondria-mediated signaling is mainly responsible for apoptosis; we further determined whether LB extract protects UVB-induced apoptosis through the regulation of mitochondrial signaling. The JC-1 fluorescence dye was used for detecting the loss of mitochondrial membrane potential (MMP), a marker of mitochondrial-mediated apoptosis [30]; therefore, we assessed the MMP membrane potential (Δ*Ψ*m) in UVB-irradiated cells using JC-1 staining. As shown in Figure 5, the results show that *L. barbarum* extract pretreatment had strong intensity of red fluorescence (J-aggregation) and weak intensity of green fluorescence (JC-1 monomer) compared to UVB irradiation alone, indicating that both *L. barbarum* extracts LBA and LBE prevent the loss of MMP in UVB-irradiated cells.

### 3.6. LBA and LBE Protected UVB-Induced DNA Damage of ARPE-19 Cells

**Figure 6:**
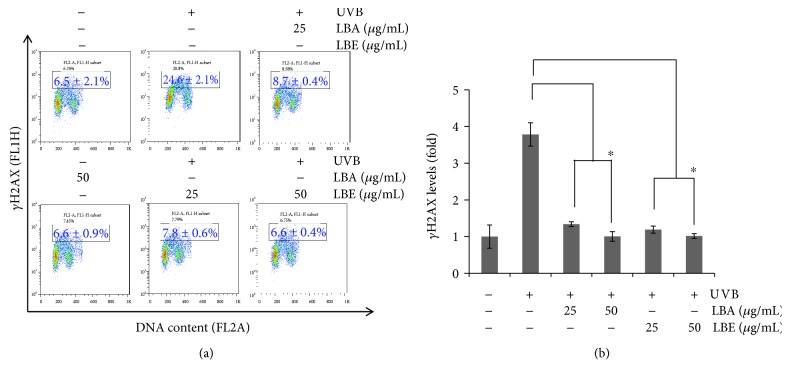
The protective effect of LB extracts on UVB-induced DNA damage of ARPE-19 cells. (a) ARPE-19 cells pretreated with LBA and LBE 25 and 50 *μ*g/mL for 2 hr prior to the exposure to 50 mJ/cm^2^ UVB irradiation, cells were harvested and subjected to the flow cytometer-based *γ*H2AX immunostaining assay and repeated in three independent experiments. (b) The quantitative analyses of *γ*H2AX level in ARPE-19 cells (*n* = 3). The asterisk indicates *p* < 0.05* vs.* UVB-exposed cells without the pretreatment of LB extracts.

*γ*H2AX, a phosphorylated histone variant H2AX at site Ser^139^, is a marker of DNA damage [31]. The assessment of *γ*H2AX is widely used for detecting DNA damage. As shown in Figure 6, the protective ability of both LBA and LBE pretreated with various concentrations at 25 and 50 *μ*g/mL shows 24.6 ± 2.1% of UVB-irradiated, 8.7 ± 0.4% and 6.6 ± 0.9% of LBA, and 7.8 ± 0.6% and 6.6 ± 0.4% of LBE significantly attenuated the activation of *γ*H2AX in a dose-responsive manner, suggesting a protective role of both LBA and LBE in UVB-induced apoptosis of human ARPE-19 cells.

## 4. Discussion

Chronic photooxidative stress from the environment could cause various damages including the integrity of the membrane, increased ROS, DNA damage, and cell death of RPE. Dysfunction and degeneration of RPE cells are crucially involved in the pathogenesis of AMD or other RPE degeneration-associated diseases [32, 33]. The apoptotic death of RPE cells, followed by photoreceptor cell death, is thought to mainly contribute to the pathogenesis of the dry form of AMD [33]. The progression of AMD pathogenesis is highly correlated with various oxidative stresses and could be prevented or delayed by supplying vitamin C or other antioxidants [34, 35]. *L. barbarum* (wolfberry) has been used as a traditional antiaging herb in Chinese pharmacopeia over a long history [36]. LBA, the primary active ingredient of lycium barbarum, isolated from the aqueous extracts of lycium barbarum, has multibioactivity properties on modulating cellular physiology, and LBA could exert protective effects on hepatocytes and neurons of the eye [37–40]. However, little is known regarding ethanol extracts of LB.

To confirm whether LB extracts LBA and LBE could protect ARPE-19 cells against UVB-induced cell damage, we first examine the viability of ARPE-19 cells after the irradiation of UVB. Our results showed that the pretreatments of both LBA and LBE significantly mitigated the proliferation of ARPE-19 cells. Under normal conditions, reactive oxygen species (ROS) could act as a second messenger in cell signaling and regulates various biological functions [25–27]; however, a high level of endogenous ROS is highly correlated with the pathophysiology of retinal degeneration diseases including AMD. Here, we demonstrated that both aqueous and ethanol extracts of *L. barbarum* could enhance antioxidant activities and attenuate the levels of endogenous ROS in ARPE-19 cells. Furthermore, both aqueous and ethanol extracts of *L. barbarum* have potent antioxidant activities and rescue cells from UVB-induced apoptosis. Moreover, ethanol extracts of *L. barbarum* display stronger antioxidant effects than the aqueous extract of *L. barbarum*. For bioactive constituents, *L. barbarum* is rich in antioxidants, including hydrophilic polysaccharides [41] and the hydrophobic flavonoids carotenoid and riboflavin [42]. Despite polysaccharide fractions, LBP has been shown to have antioxidant activity, and a previous study revealed that crude polysaccharide extracts exhibited stronger antioxidant activity than purified polysaccharide fractions (LBP) because antioxidants such as carotenoids, riboflavin, ascorbic acid, thiamine, and nicotinic acid are more abundant in crude extracts. In our study, the results showed that both LBA and LBE reduced the level of ROS in human ARPE-19 cells after UVB irradiation. Moreover, the JC-1 staining for detecting the loss of mitochondrial membrane potential (MMP) as a marker of mitochondrial-mediated apoptosis was conducted, and the results showed that the pretreatment of both LB extracts prevented the loss of MMP following UVB irradiation.

Therefore, we further determined whether LBA and LBE occluded cell apoptosis under UVB-irradiated ARPE-19 cells using a cytometer-based Annexin V/PI double staining assay. Consistently, our results showed that UVB-induced apoptosis of ARPE-19 cell at 24 hr; however, the percentages of UVB-induced apoptotic cells were decreased with both LB extract pretreatments. Both extracts exerted protective effects on UVB-induced apoptosis in an ARPE-19 cell line. It is well known that irradiation of UVB or visible light causes photochemical lesions or other damages through the induction of DNA damage [43]; therefore, examination of whether LB extracts could protect ARPE-19 cells from DNA damage using the assessment of *γ*H2AX, a marker of DNA damage [31], is desirable.

Our results showed that the pretreatments of both LBE and LBA significantly attenuated the activation of *γ*H2AX in a dose-responsive manner, suggesting a protective role of LB extracts in UVB-induced DNA damage of human ARPE-19 cells. Because of the great benefits of *L. barbarum*, many studies have been conducted extensively on the preventive effects of LB extracts against eye diseases [44–46]. For example, the work of Li et al. suggested that LBP is neuroprotective and could delay secondary degeneration of retinal ganglion cells (RGCs), which might be associated with the downregulation of oxidative stress and the MAPK JNK/c-Jun pathway in retinal tissue [46]. Furthermore, the work of Liu et al. demonstrated that the treatment of LBP not only improves morphology and function of retinal tissue in rd1 mice, an *in vivo* photoreceptor degenerating model of retinitis pigmentosa, but also delays the functional degeneration of RGCs [45]. However, little is known regarding the bioactivity and mechanism of *L. barbarum* against DNA damage; consequently, our study is the first to reveal *L. barbarum* extracts as protective agents for UVB-induced DNA damage.

**Figure 7:**
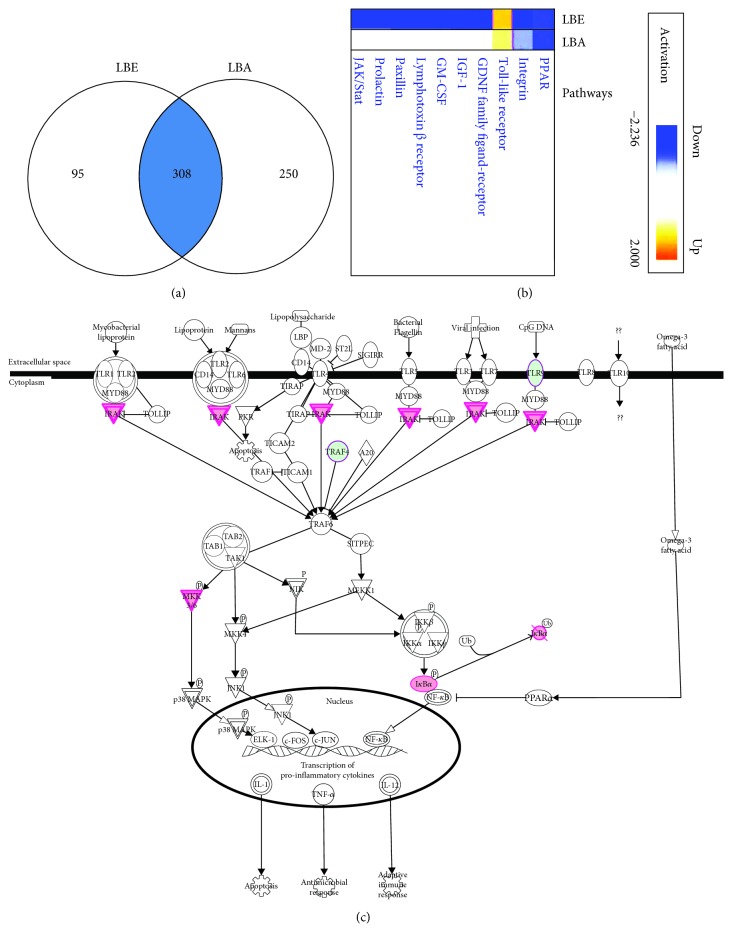
Activation of genes involved in *L. barbarum* extract-mediated protective effect on UVB irradiation. The human global gene expression profiles of RNA were isolated from ARPE-19 cells pretreated with *L. barbarum* extracts LBE or LBA prior to the irradiation of UVB then analyzed by a cDNA microarray at 24 hr. (a) A total of 403 genes in LBE and a total of 558 genes in LBA were changed, and 308 genes might be involved in the signaling pathways associated with the protective effect of *L. barbarum* extracts using the analysis of IPA bioinformatics software. (b). Top-level functional annotation categories of genes including peroxisome proliferator-activated receptor (PPAR), the nuclear receptor as a transcription factor, Integrin, and Toll-like receptor (TLR) signaling pathways were affected by the pretreatment of both *L. barbarum* extracts LBE and LBA. Remarkably, the activation level of the TLR pathway is higher in the LBE pretreatment than in the LBA pretreatment. (c) Genes might be involved in the activation of the TLR pathway. A pink color indicates the upregulation of the genes. A green color indicates the downregulation of the genes.

Previously, Lin et al.'s work analyzed the interrelationship between *Lycium barbarum* and gene expression in mouse spleen using oligo-microarray, and their result showed that three genes, Bcl-2, NF*κ*B, and TNF, were upregulated whereas two proapoptotic genes (apoptotic protease-activating factor-1 (apaf-1) and caspase-3) were downregulated by the treatment of *Lycium barbarum* [47]. Given the superior cytoprotection on UVB irradiation-induced growth arrest by ethanol extract LBE, we further investigated the mechanism using human global gene expression profiles of RNA isolated from ARPE-19 cells pretreated with *L. barbarum* extracts LBE or LBA prior to the irradiation of UVB analyzed by a cDNA microarray at 24 hr. We identified that a total of 403 genes in LBE-treated cells and a total of 558 genes in LBA-treated cells were changed, and 308 genes might be involved in the signaling pathways associated with the protective effect of *L. barbarum* extracts using analysis by IPA bioinformatics software (Figure 7(a)). The analysis of IPA further suggested that three signaling pathways of the top-level functional annotation categories of genes including PPAR, Integrin survival signaling, and Toll-like receptor (TLR) signaling pathways were affected by the pretreatment of both *L. barbarum* extracts LBE and LBA (Figures 7(b) and 7(c)).

Peroxisome proliferator-activated receptors (PPARs) are important nuclear transcription factors for oxidative defense system in eukaryotic cells. The PPAR family has at least three members including PPAR*α*, PPAR*β*/*δ*, and PPAR*γ* [48]. For example, PPAR*γ* has been shown to have an antioxidant function through transcriptional regulating a number of antioxidation-associated gene catalase (CAT), glutathione peroxidase (GPX-3), heme oxygenase-1 (HO-1), and manganese superoxide dismutase (MnSOD) [49].

Integrins are transmembrane proteins and the heterodimerization of integrin receptors have been shown to regulate cell survival, differentiation, and migration of metazoa through communicating signals via the plasma membrane [50]. Neuronal survival is exhibited by olfactory ensheathing cell, a type of glia that supports axon outgrowth olfactory system through integrin/milk fat globule-EGF factor 8 ((MFG-E8) a secreted glycoprotein) signaling pathway [51]. Moreover, a recent study demonstrated that the sensory axons of the spinal cord can regenerate by expressing tenascin-binding *α*9-integrin together with kindlin-1, the integrin activator [52].

Toll-like receptors (TLRs) are single and noncatalytic receptors with membrane-spanning domains and the TLRs can recognize microbial pathogen and initiate signal transduction pathways of innate immunity [53]. Beyond the role of TLRs in activating innate immunity, TLR seems to play a critical role in neuron survival [54–57]. Patel and Hackam's work suggested that the oxidative stress-activated TLR3 triggers neuron protection rather than pathogenic signaling in the retina tissue of mice [58]. Bsibsi et al.'s work showed that inflammation-induced TLR-3 activation triggers a neuroprotective response rather than a proinflammatory response in human astrocytes [59]. Furthermore, Jeong et al.'s work showed that polyinosinic poly-cytidylic acid- (poly(I:C)-) induced activation of TLR3 might favor the survival of microglia after cerebral ischemia, suggesting the neuroprotective role of TLR3 [60]. Controversially, the activation of TLR might also create a degenerative effect in neuron cells; for example, Chintala et al.'s work showed that poly(I:C)-induced TLR3 activation upregulated the protein level of MAPK JNK3 and promoted the degeneration of RGCs in mouse eye [61]. Similarly, Gao et al.'s work showed that poly(I:C)-induced TLR3 evoked an inflammatory response and cell death both in murine photoreceptor 661W cells and an *in vivo* model [62]. Therefore, the role of TLR in prosurvival or pro-cell death of neuron cells may depend on the types [55] of stimuli, the level [60] of stimuli, and the downstream of TLR signaling [58].

In the results of microarray analysis combining the IPA bioinformatics approach, the activation level of the TLR pathway is higher in LBE pretreatment than that in LBA pretreatment (Figures 7(b) and 7(c)). Given the pivotal role of the TLR pathway in cell proliferation and cell survival [63], it is worth further investigating the three signaling pathways, especially the TLR pathway in LB extract-mediated cytoprotection in human retinal pigment epithelial cells.

### 4.1. LBA and LBE Rescue UVB-Induced G_2_-Arrest after Irradiated ARPE-19

In the study, 50 mJ/cm^2^ of UVB irradiation significantly caused the accumulation of cell cycle G_2_/M population in human ARPE-19 cells. Interestingly, Chou et al.'s work showed that 10 to 30 mJ/cm^2^ UVB caused S-arrest in ARPE-19 cells [64]; we suggest that different doses of UVB irradiation might result in the discrepancy in the arrest of cell cycle S and G_2_/M phases. Furthermore, the pretreatment of both LBA and LBE rescues UVB irradiation-induced G_2_/M-arrest in ARPE-19 (Figure S1).

## 5. Conclusions

**Figure 8:**
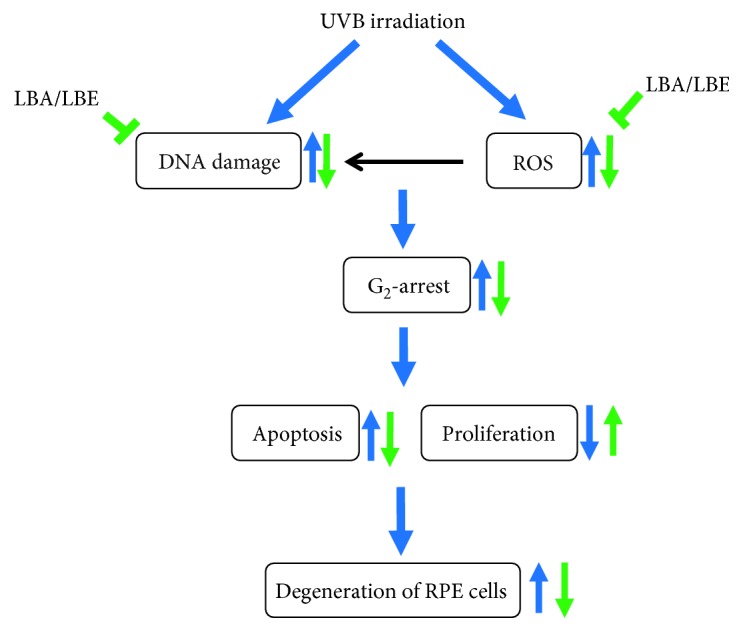
The proposed mechanism of *Lycium barbarum* extract-mediated protection against UVB-induced damage in human retinal pigment epithelial (RPE) cells. The irradiation of UVB causes the increase of endogenous ROS and the induction of DNA damage, resulting in the damage and degeneration of RPE cells. The extracts of *L. barbarum* (wolfberry) LBA and LBE exert the potent antioxidant activity and attenuate the DNA damage induced by UVB irradiation in RPE cells, therefore, protecting the RPE cells from UVB-induced cell cycle G_2_/M-phase arrest, growth inhibition, and apoptosis, further delaying or attenuating the degeneration of retinal pigment epithelial cells. The blue arrows indicate the impacts of UVB exposure. Green arrows indicate the bioactivities of *L. barbarum* extracts.

We demonstrated that both aqueous and ethanol extracts of *L. barbarum* exhibit antioxidant activities and prevent UVB irradiation-induced DNA damage and apoptosis of ARPE cells. Interestingly, ethanol extracts of *L. barbarum* exert stronger antioxidant than aqueous extract, suggesting that polyphenolic components might enhance the antioxidant activities of the ethanol extract of *L. barbarum*. Based on the results of the present study, we conclude that both aqueous and ethanol extracts of *L. barbarum* protect ARPE-19 cells from UVB exposure-induced growth and apoptosis through reducing endogenous ROS and DNA damage (Figure 8). This study is the first to depict the cytoprotective activity of *L. barbarum* extracts on cytoprotection in UVB-induced DNA damage in ARPE-19 retinal cells that might be correlated with three signaling pathways including PPAR, Integrin, and TLR. Further investigation on the ethanol extracts of *L. barbarum* will benefit the prevention of retinal degenerative-associated diseases such as AMD in the future.

## Data Availability

The data used to support the findings of this study are available from the corresponding author upon request.

## Supplementary Materials

Supplementary Materials Figure S1: the effect of *L. barbarum* extract pretreatments on UVB-induced G2-arrest in ARPE-19 cells. ARPE-19 cells were pretreated with 50 *μ*g/mL *L. barbarum* extracts LBA or LBE, respectively, for 2 hr prior to the irradiation of UVB described in the section of Materials and Methods.

## References

[1] Macular Photocoagulation Study Group, Maguire M. G., Fine S. L. (1994). Visual outcome after laser photocoagulation for subfoveal choroidal neovascularization secondary to age-related macular degeneration: the influence of initial lesion size and initial visual-acuity. *Archives of Ophthalmology*.

[2] Nilsson S. E. G., Sundelin S. P., Wihlmark U., Brunk U. T. (2003). Aging of cultured retinal pigment epithelial cells: oxidative reactions, lipofuscin formation and blue light damage. *Documenta Ophthalmologica*.

[3] Lim T. K., Lim T. K. (2013). Lycium barbarum. *Edible Medicinal and Non-Medicinal Plants: Volume 6, Fruits*.

[4] Capetandes A., Gerritsen M. E. (1990). Simplified methods for consistent and selective culture of bovine retinal endothelial cells and pericytes. *Investigative Ophthalmology and Visual Science*.

[5] Yue B., Kawa J., Chang I., Sawaguchi S., Fishman G. (1991). Effects of chondroitin sulfate on cultured human retinal pigment epithelial cells. *Cell Biology International Reports*.

[6] Tang J., Kern T. S. (2011). Inflammation in diabetic retinopathy. *Progress in Retinal and Eye Research*.

[7] Sivak J. M., Fini M. E. (2002). MMPs in the eye: emerging roles for matrix metalloproteinases in ocular physiology. *Progress in Retinal and Eye Research*.

[8] Zheng Z., Chen H., Ke G. (2009). Protective effect of perindopril on diabetic retinopathy is associated with decreased vascular endothelial growth factor-to-pigment epithelium-derived factor ratio: involvement of a mitochondria-reactive oxygen species pathway. *Diabetes*.

[9] Cheung N., Mitchell P., Wong T. Y. (2010). Diabetic retinopathy. *Lancet*.

[10] Dupont C., Armant D., Brenner C. (2009). Epigenetics: definition, mechanisms and clinical perspective. *Seminars in Reproductive Medicine*.

[11] Miller-Kasprzak E., Jagodzinski P. P. (2008). 5-Aza-2′-deoxycytidine increases the expression of anti-angiogenic vascular endothelial growth factor 189b variant in human lung microvascular endothelial cells. *Biomedicine and Pharmacotherapy*.

[12] Chabane N., Zayed N., Afif H. (2008). Histone deacetylase inhibitors suppress interleukin-1beta-induced nitric oxide and prostaglandin E2 production in human chondrocytes. *Osteoarthritis and Cartilage*.

[13] Gao G., Li Y., Zhang D., Gee S., Crosson C., Ma J. X. (2001). Unbalanced expression of VEGF and PEDF in ischemia-induced retinal neovascularization. *FEBS Letters*.

[14] Pons M., Marin-Castano M. E. (2011). Nicotine increases the VEGF/PEDF ratio in retinal pigment epithelium: a possible mechanism for CNV in passive smokers with AMD. *Investigative Ophthalmology and Visual Science*.

[15] Gao G., Li Y., Fant J., Crosson C. E., Becerra S. P., Ma J. X. (2002). Difference in ischemic regulation of vascular endothelial growth factor and pigment epithelium--derived factor in brown Norway and Sprague Dawley rats contributing to different susceptibilities to retinal neovascularization. *Diabetes*.

[16] Bharadwaj A. S., Appukuttan B., Wilmarth P. A. (2013). Role of the retinal vascular endothelial cell in ocular disease. *Progress in Retinal and Eye Research*.

[17] Simo R., Villarroel M., Corraliza L., Hernandez C., Garcia-Ramirez M. (2010). The retinal pigment epithelium: something more than a constituent of the blood-retinal barrier--implications for the pathogenesis of diabetic retinopathy. *Journal of Biomedicine & Biotechnology*.

[18] Elner V. M., Scales W., Elner S. G., Danforth J., Kunkel S. L., Strieter R. M. (1992). Interleukin-6 (IL-6) gene expression and secretion by cytokine-stimulated human retinal pigment epithelial cells. *Experimental Eye Research*.

[19] Broadhead M. L., Becerra S. P., Choong P. F. M., Dass C. R. (2010). The applied biochemistry of PEDF and implications for tissue homeostasis. *Growth Factors*.

[20] Liu X., Chen H. H., Zhang L. W. (2013). Potential therapeutic effects of pigment epithelium-derived factor for treatment of diabetic retinopathy. *International Journal of Ophthalmology*.

[21] Cui B. K., Liu S., Lin X. J. (2011). Effects of Lycium barbarum aqueous and ethanol extracts on high-fat-diet induced oxidative stress in rat liver tissue. *Molecules*.

[22] Chiu C. C., Liu P. L., Huang K. J. (2011). Goniothalamin inhibits growth of human lung cancer cells through DNA damage, apoptosis, and reduced migration ability. *Journal of Agricultural and Food Chemistry*.

[23] Yen C. Y., Chiu C. C., Haung R. W. (2012). Antiproliferative effects of goniothalamin on Ca9-22 oral cancer cells through apoptosis, DNA damage and ROS induction. *Mutation Research*.

[24] Kan J. Y., Yen M. C., Wang J. Y. (2016). Nesfatin-1/nucleobindin-2 enhances cell migration, invasion, and epithelial-mesenchymal transition via LKB1/AMPK/TORC1/ZEB1 pathways in colon cancer. *Oncotarget*.

[25] D'Autreaux B., Toledano M. B. (2007). ROS as signalling molecules: mechanisms that generate specificity in ROS homeostasis. *Nature Reviews Molecular Cell Biology*.

[26] Ushio-Fukai M., Nakamura Y. (2008). Reactive oxygen species and angiogenesis: NADPH oxidase as target for cancer therapy. *Cancer Letters*.

[27] Clerkin J. S., Naughton R., Quiney C., Cotter T. G. (2008). Mechanisms of ROS modulated cell survival during carcinogenesis. *Cancer Letters*.

[28] Geest C. R., Buitenhuis M., Groot Koerkamp M. J. A., Holstege F. C. P., Vellenga E., Coffer P. J. (2009). Tight control of MEK-ERK activation is essential in regulating proliferation, survival, and cytokine production of CD34+-derived neutrophil progenitors. *Blood*.

[29] Wu X., Hua X. (2014). Targeting ROS: selective killing of cancer cells by a cruciferous vegetable derived pro-oxidant compound. *Cancer Biology & Therapy*.

[30] Li H. H., Su J. H., Chiu C. C. (2013). Proteomic investigation of the sinulariolide-treated melanoma cells A375: effects on the cell apoptosis through mitochondrial-related pathway and activation of caspase cascade. *Marine Drugs*.

[31] Valdiglesias V., Giunta S., Fenech M., Neri M., Bonassi S. (2013). γH2AX as a marker of DNA double strand breaks and genomic instability in human population studies. *Mutation Research*.

[32] Roth F., Bindewald A., Holz F. G. (2004). Keypathophysiologic pathways in age-related macular disease. *Graefes Archive for Clinical and Experimental Ophthalmology*.

[33] Nowak J. Z. (2006). Age-related macular degeneration (AMD): pathogenesis and therapy. *Pharmacological Reports*.

[34] The Age-Related Eye Disease Study 2 (AREDS2) Research Group (2013). Lutein + zeaxanthin and omega-3 fatty acids for age-related macular degeneration: the Age-Related Eye Disease Study 2 (AREDS2) randomized clinical trial. *JAMA*.

[35] Age-Related Eye Disease Study Research Group (2001). A randomized, placebo-controlled, clinical trial of high-dose supplementation with vitamins C and E, beta carotene, and zinc for age-related macular degeneration and vision loss: AREDS report no. 8. *Archives of Ophthalmology*.

[36] Ho Y.-S., So K.-F., Chang R. (2011). Drug discovery from Chinese medicine against neurodegeneration in Alzheimer’s and vascular dementia. *Chinese Medicine*.

[37] Chan H. C., Chuen-Chung Chang R., Koon-Ching Ip A. (2007). Neuroprotective effects of Lycium barbarum Lynn on protecting retinal ganglion cells in an ocular hypertension model of glaucoma. *Experimental Neurology*.

[38] Chiu K., Chan H. C., Yeung S. C. (2009). Modulation of microglia by wolfberry on the survival of retinal ganglion cells in a rat ocular hypertension model. *Journal of Ocular Biology, Diseases, and Informatics*.

[39] Chiu K., Zhou Y., Yeung S. C. (2010). Up-regulation of crystallins is involved in the neuroprotective effect of wolfberry on survival of retinal ganglion cells in rat ocular hypertension model. *Journal of Cellular Biochemistry*.

[40] Li S. Y., Yang D., Yeung C. M. (2011). Lycium barbarum polysaccharides reduce neuronal damage, blood-retinal barrier disruption and oxidative stress in retinal ischemia/reperfusion injury. *PLoS One*.

[41] Ming M., Guanhua L., Zhanhai Y., Guang C., Xuan Z. (2009). Effect of the *Lycium barbarum* polysaccharides administration on blood lipid metabolism and oxidative stress of mice fed high-fat diet in vivo. *Food Chemistry*.

[42] Luo Q., Cai Y., Yan J., Sun M., Corke H. (2004). Hypoglycemic and hypolipidemic effects and antioxidant activity of fruit extracts from Lycium barbarum. *Life Sciences*.

[43] Sparrow J. R., Nakanishi K., Parish C. A. (2000). The lipofuscin fluorophore A2E mediates blue light-induced damage to retinal pigmented epithelial cells. *Investigative Ophthalmology and Visual Science*.

[44] Tang L., Bao S., du Y. (2018). Antioxidant effects of *Lycium barbarum* polysaccharides on photoreceptor degeneration in the light-exposed mouse retina. *Biomedicine and Pharmacotherapy*.

[45] Liu F., Zhang J., Xiang Z. (2018). Lycium barbarum polysaccharides protect retina in rd 1 mice during photoreceptor degeneration. *Investigative Ophthalmology and Visual Science*.

[46] Li H., Liang Y., Chiu K. (2013). Lycium barbarum (wolfberry) reduces secondary degeneration and oxidative stress, and inhibits JNK pathway in retina after partial optic nerve transection. *PLoS One*.

[47] Lin N. C., Lin J. C., Chen S. H., Ho C. T., Yeh A. I. (2011). Effect of goji (Lycium barbarum) on expression of genes related to cell survival. *Journal of Agricultural and Food Chemistry*.

[48] Agarwal S., Yadav A., Chaturvedi R. K. (2017). Peroxisome proliferator-activated receptors (PPARs) as therapeutic target in neurodegenerative disorders. *Biochemical and Biophysical Research Communications*.

[49] Lee C. (2017). Collaborative power of Nrf 2 and PPARγ activators against metabolic and drug-induced oxidative injury. *Oxidative Medicine and Cellular Longevity*.

[50] Lau T. L., Kim C., Ginsberg M. H., Ulmer T. S. (2009). The structure of the integrin αIIbβ3 transmembrane complex explains integrin transmembrane signalling. *EMBO Journal*.

[51] Li Y., Zou T., Xue L., Yin Z. Q., Huo S., Xu H. (2017). TGF-β1 enhances phagocytic removal of neuron debris and neuronal survival by olfactory ensheathing cells via integrin/MFG-E8 signaling pathway. *Molecular and Cellular Neuroscience*.

[52] Fawcett J. W. (2017). An integrin approach to axon regeneration. *Eye*.

[53] Takeda K., Akira S. (2005). Toll-like receptors in innate immunity. *International Immunology*.

[54] Dvoriantchikova G., Barakat D. J., Hernandez E., Shestopalov V. I., Ivanov D. (2010). Toll-like receptor 4 contributes to retinal ischemia/reperfusion injury. *Molecular Vision*.

[55] Lehnardt S., Massillon L., Follett P. (2003). Activation of innate immunity in the CNS triggers neurodegeneration through a Toll-like receptor 4-dependent pathway. *Proceedings of the National Academy of Sciences of the United States of America*.

[56] Yang Z., Stratton C., Francis P. J. (2008). Toll-like receptor 3 and geographic atrophy in age-related macular degeneration. *New England Journal of Medicine*.

[57] Hanisch U. K., Johnson T. V., Kipnis J. (2008). Toll-like receptors: roles in neuroprotection?. *Trends in Neurosciences*.

[58] Patel A. K., Hackam A. S. (2014). A novel protective role for the innate immunity Toll-like receptor 3 (TLR3) in the retina via stat 3. *Molecular and Cellular Neuroscience*.

[59] Bsibsi M., Persoon-Deen C., Verwer R. W. H., Meeuwsen S., Ravid R., van Noort J. M. (2006). Toll-like receptor 3 on adult human astrocytes triggers production of neuroprotective mediators. *Glia*.

[60] Jeong S. Y., Jeon R., Choi Y. K. (2016). Activation of microglial Toll-like receptor 3 promotes neuronal survival against cerebral ischemia. *Journal of Neurochemistry*.

[61] Chintala S. K., Putris N., Geno M. (2015). Activation of TLR3 promotes the degeneration of retinal ganglion cells by upregulating the protein levels of JNK3. *Investigative Ophthalmology and Visual Science*.

[62] Gao M.-L., Wu K.-C., Deng W.-L. (2017). Toll-like receptor 3 activation initiates photoreceptor cell death in vivo and in vitro. *Investigative Opthalmology & Visual Science*.

[63] Li X., Jiang S., Tapping R. I. (2010). Toll-like receptor signaling in cell proliferation and survival. *Cytokine*.

[64] Chou W. W., Chen K. C., Wang Y. S., Wang J. Y., Liang C. L., Juo S. H. H. (2013). The role of SIRT1/AKT/ERK pathway in ultraviolet B induced damage on human retinal pigment epithelial cells. *Toxicology In Vitro*.

