# Supplementary figures and images for: Protective Effects of Lycium barbarum Extracts on UVB-Induced Damage in Human Retinal Pigment Epithelial Cells Accompanied by Attenuating ROS and DNA Damage
Source: Oxid Med Cell Longev. 2018 Nov 7;2018:4814928. doi: 10.1155/2018/4814928 (PMC6247443; doi:10.1155/2018/4814928)

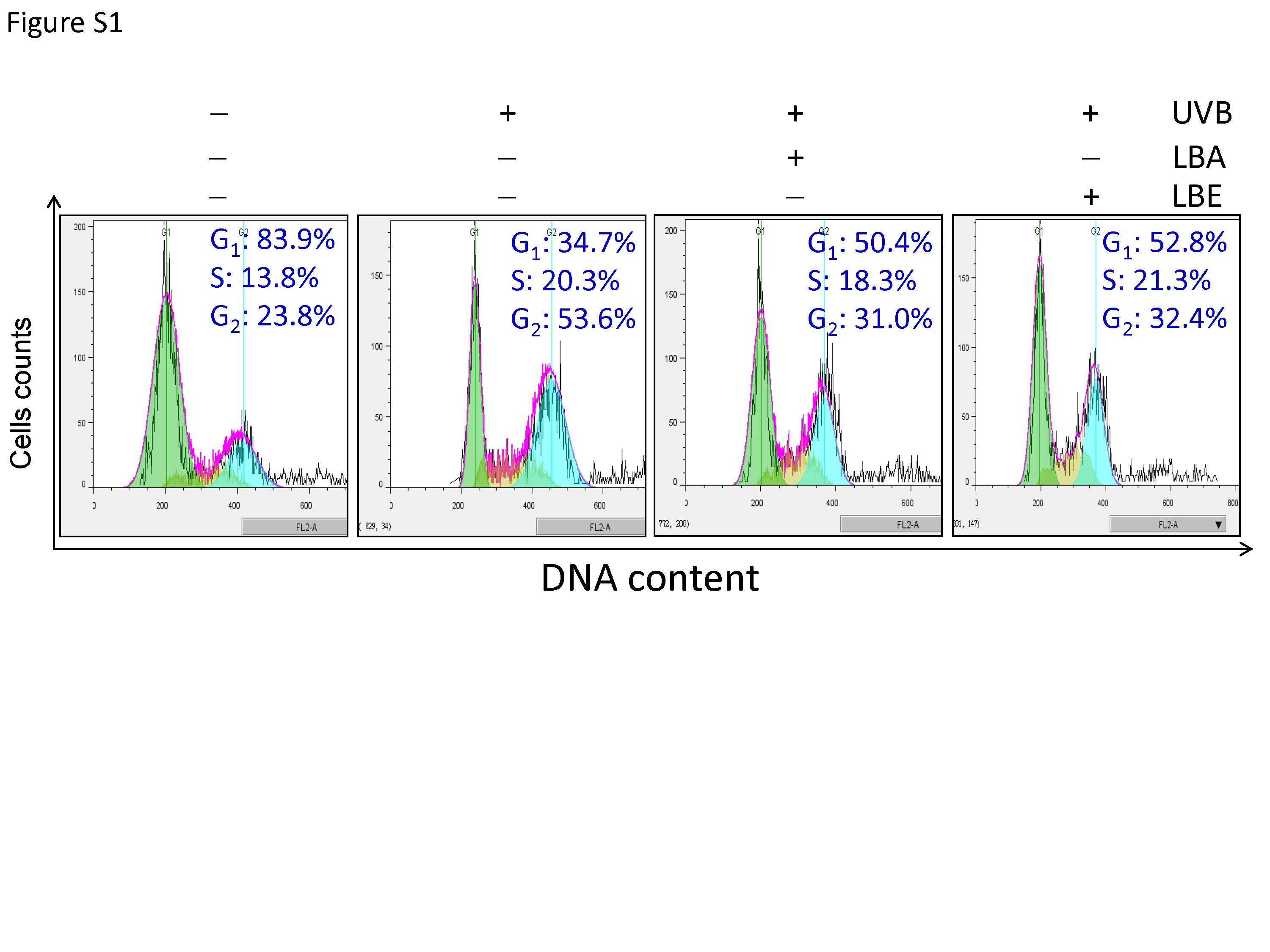

Supplement: Supplementary Materials — Figure S1: the effect of L. barbarum extract pretreatments on UVB-induced G2-arrest in ARPE-19 cells. ARPE-19 cells were pretreated with 50 μg/mL L. barbarum extracts LBA or LBE, respectively, for 2 hr prior to the irradiation of UVB described in the section of Materials and Methods. [file 4814928.f1.tiff]
